# Effect of RISCO–NUTRIFOUR Probiotic on Growth and Carcass Traits in Broilers During Starter and Grower Phases

**DOI:** 10.1002/fsn3.71335

**Published:** 2026-09-18

**Authors:** Abdulaziz A. Al‐abdullatif, Maged A. Al‐Garadi, Mohammed M. Qaid, Abdulkareem M. Matar, Mohammed Al‐Badwi, Mohsen M. Alobre, Gamaleldin M. Suliman, Elsayed O. Hussein

**Affiliations:** ^1^ Department of Animal Production, College of Food and Agricultural Sciences King Saud University Riyadh Saudi Arabia

**Keywords:** *B. subtilis*, broilers, carcass traits, performance indices, RISCO–NUTRIFOUR probiotics, *S. cerevisiae*

## Abstract

The growing concern over antibiotic resistance has prompted the exploration of probiotics as sustainable alternatives in poultry production. This study evaluated the effects of a multi‐strain, multi‐genera probiotic supplement (RISCO‐NUTRIFOUR solution (RNFS), administered via drinking water (Days 1–14) and feed (Days 15–28)) on broiler performance and carcass characteristics over a 28‐day period. A total of 288 day‐old Ross 308 chicks were randomly allocated to six treatments: T0—no probiotic supplementation (0.0% RNFS); T1‐T3: RNFS at 0.1%, 0.2%, and 0.4%, respectively; T4: *Bacillus subtilis* ( *B. subtilis* ) at 0.1%; and T5: *Saccharomyces cerevisiae* ( *S. cerevisiae* ) at 0.1%. Body weight gain (BWG), daily feed intake (DFI), and feed conversion ratio (FCR) were recorded during Days 0–14, 14–28, and cumulatively (0–28). On Day 28, relative weights of breast, legs, wings, proventriculus, heart, kidney, pancreas, liver, and lymphoid organs (thymus, spleen, bursa of Fabricius) were also measured. Broilers receiving RNFS, particularly at the 0.2% level, showed significant improvements in final average body weight and BWG during the grower phase (Days 14–28), compared to the control groups. Carcass traits were largely unaffected, except for a significant increase (22.54%) in gizzard weight in the 0.2% RNFS group. In conclusion, RNFS supplementation at 0.2% proved most effective, significantly enhancing growth performance and numerically improving feed efficiency in broilers, supporting its potential as a practical alternative to single‐strain probiotics.

## Graphical abstract

Effect of RISCO–NUTRIFOUR Probiotic on Growth and Carcass Traits in Broilers During Starter and Grower Phases. A multi‐strain, multi‐kingdom probiotic (RISCO–NUTRIFOUR) administered through drinking water during the starter phase and through feed during the grower phase markedly enhanced broiler growth performance and feed conversion. Dose‐dependent improvements were observed, with 0.2%–0.4% RNFS outperforming single‐strain probiotics and demonstrating its efficacy as an early‐life performance enhancer in broilers.

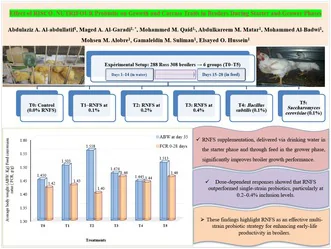

## 1 Introduction

Efficient nutrient absorption is fundamental to optimizing growth performance and maintaining welfare in poultry production. Traditionally, antibiotics have been widely used as growth promoters to enhance gut health and nutrient utilization (El‐Sayed et al. 2024; Magnoli et al. 2024). However, increasing concerns over antimicrobial resistance and the associated risk of horizontal gene transfer to the human microbiome have driven global regulatory restrictions, including the European Union's ban on antibiotic growth promoters (Regulation (EC) No. 1831/2006). This shift has intensified interest in alternatives such as probiotics, which can support sustainable productivity and improved health in broiler chickens.

Poultry gut health is multifaceted and depends on efficient digestion and nutrient absorption, a well‐balanced microbiota, intact mucosal barriers, and regulated immune and neuroendocrine responses (Gao et al. 2025; Pluske et al. 2018). Microbial dysbiosis may predispose birds to enteric infections, feed inefficiency, and economic loss (Karadedos et al. 2025). Probiotics—live, non‐pathogenic microorganisms that confer health benefits to the host—support gut health by improving mucosal integrity, modulating intestinal microflora, enhancing nutrient uptake, and strengthening immune responses (Farooq 2024; Kushwaha and Maurya 2024; Mishra and Acharya 2021; Rashid et al. 2023). Probiotics can be administered through either drinking water or feed, with both routes offering distinct advantages (Hossain et al. 2025; Izadi et al. 2025). Several studies further demonstrate improved villus architecture and feed efficiency as a result of probiotic supplementation (Abd El‐Hack et al. 2022; Attia et al. 2023; Shaw et al. 2024; Smolinska et al. 2025; Yang et al. 2024). Consequently, probiotics have become widely accepted as practical and sustainable alternatives to antibiotic growth promoters in broiler production (Herich et al. 2025; Liu et al. 2022; Muneeb et al. 2025; Poloni et al. 2020; Reuben et al. 2021; Shehata et al. 2022).

Among probiotic species, *B. subtilis* and *S. cerevisiae* are commonly used but often yield inconsistent results, attributed to differences in dosage, strain specificity, administration route, and production conditions (Idowu et al. 2025; Istiteh et al. 2025; Soren et al. 2024). Multi‐strain formulations, however, may offer broader functional benefits through synergistic microbial interactions (Adli et al. 2025; Sardar et al. 2025; Younas et al. 2025). One such formulation is RISCO–NUTRIFOUR solution (RNFS), a multi‐strain, multi‐genera probiotic containing *B. subtilis* , *S. cerevisiae* , lactic acid bacteria, Rhodopseudomonas spp., and *Candida ethanolic*. The combined activity of bacteria, yeast, and phytochemical components has the potential to improve gut ecology, nutrient utilization, antioxidant status, and carcass quality (Osman et al. 2025; Sardar et al. 2025).

The present study builds directly upon our earlier investigations evaluating RNFS supplementation in broiler chickens. In our 2025 publication Al‐abdullatif et al. (2025c), we assessed RNFS administered via drinking water during Days 1–14, followed by dietary inclusion from Days 15–28, and examined its effects on the technological and physicochemical quality of breast meat at 28 days of age. That study demonstrated that 0.4% RNFS produced the most desirable texture profile, 0.2% optimized juiciness, and overall sensory acceptability, while 0.1% resulted in the most tender texture and lowest cooking loss compared with controls. Extending these findings, Al‐abdullatif et al. (2025b) subsequently showed that RNFS supplementation—particularly at 0.1%—also improved meat quality at 42 days without negatively affecting sensory attributes.

Additionally, our earlier experiment demonstrated that RNFS supplementation as a feed additive over a 28‐day period enhanced broiler growth performance and numerically improved carcass traits, establishing foundational evidence for the biological efficacy of the formulation (Al‐abdullatif et al. 2025d). More recently (Al‐abdullatif et al. 2025a) reported that dietary RNFS, particularly at 1 mL kg^−1^, mitigated heat‐stress‐induced detriments in meat quality and growth performance during 29–42 days, resulting in improved production index, European Production Efficiency Factor, and feed efficiency. Collectively, these sequential studies highlight the progressive development of RNFS research and support the formulation as a practical, antibiotic‐free strategy for broiler production, particularly under hot‐climate conditions.

Given the increasing interest in multi‐strain probiotics and the limited evidence on combined water‐and‐feed supplementation strategies, the present study was designed to evaluate the effects of graded levels of RNFS administered via drinking water during Days 1–14 and via feed during Days 15–28 on broiler growth performance and carcass characteristics. This work aims to address key gaps in the literature by providing new insights into dose–response patterns, early‐life physiological responses, and the comparative efficacy of RNFS relative to widely used single‐strain probiotics.

## 2 Materials and Methods

### 2.1 Ethical Approval and ARRIVE Compliance

This experiment was conducted in accordance with the ARRIVE guidelines, ensuring transparency in allocation, housing, husbandry, sample size, and outcome measures. Ethical approval was obtained from the Institutional Animal Care and Use Committee (IACUC) at King Saud University, Scientific Research Ethics Committee, KSU‐SE‐21‐47.

### 2.2 Experimental Design and Housing

A total of 288 one‐day‐old male Ross 308 broiler chicks were acquired from Al Khumasia Company for Feed and Animal Products, Riyadh, Saudi Arabia. Upon arrival, chicks were individually weighed to ensure uniform weight distribution and randomly assigned to one of six treatment groups using a random number generator in Microsoft Excel. Each group had eight replicate cages containing six birds each.

Birds were housed in climate‐controlled wire‐floor battery cages (58 × 50 × 35 cm, six birds per 0.30 m^2^) without bedding. Heating was supplied by thermostatically controlled electric brooders integrated into the climate‐control system. Environmental conditions included an initial temperature of 35°C on Day 1, gradually decreased by 2°C every 3 days until Day 24, and kept constant thereafter, with relative humidity maintained around 65%. Lighting was continuous (24 h) during the first week and then reduced to 23 h on/1 h off thereafter. Outdoor ambient temperature averaged 26.4°C with mild‐to‐moderate humidity. All birds received the standard vaccinations schedule against IBD, NDV, and IBV (Fort Dodge Animal Health, USA) via spray at hatchery on Day 0.

### 2.3 Treatments and Dietary Management

Birds were randomly assigned to six treatments, administered via drinking water during Days 0–14 and incorporated into feed during Days 15–28. The treatments included: T0: a negative control group receiving only the basal diet without probiotic supplementation. T1–T3: RNFS at three inclusion levels 0.1%, 0.2%, and 0.4%, corresponding to 1, 2, and 4 mL/kg feed, respectively; T4: a single‐strain *B. subtilis* probiotic (CloSTAT, 0.1%; Kemin Industries); a yeast probiotic; and T5: *S. cerevisiae* (SafMannan, 0.1%; Phileo, Lesaffre, France).

The RISCO‐NUTRIFOUR probiotic contained a multi‐strain multi‐genera blend: *B. subtilis* (1 × 10^9^ Colony forming units (CFU)/ml), *S. cerevisiae* yeast (1 × 10^5^ CFU/mL), *Lactobacillus harbinensis* and *Lactobacillus parabunchneri* (1 × 10^9^ CFU/mL each), *Rhodopseudomonas palustris* and *Rhodopseudomonas shaeroides* (1 × 10^7^ CFU/mL each), and *Candida ethanolic* (1 × 10^5^ CFU/mL). This product was supplied by Al Raya Specialties, Riyadh, Saudi Arabia. The RNFS strains were selected based on prior validation, which included Bacillus, Saccharomyces, Lactobacillus, and Rhodopseudomonas species previously incorporated at 0.1% (W/W) in a basal poultry diet (Bostami et al. 2016).

Basal diets in mash form were formulated according to Aviagen's Ross 308 nutrition specifications, for both starter (1–14 days) and grower (15–28 days) phases (see Table 1). Birds had *ad libitum* access to feed and water throughout the study.

**TABLE 1:** Feed components and nutritional content of basal diets (%).

Ingredients	Starter	Grower
Corn Grain	53.92	55.51
Soybean Meal 48	38.23	35.90
Soybean Oil	3.44	4.65
Limestone	1.54	1.39
Common Salt	0.38	0.38
Vitamin Premix^a^	0.10	0.10
Mineral Premix^b^	0.10	0.10
DL‐Methionine	0.35	0.31
L‐Lysin HCL	0.21	0.15
L‐Threonine	0.14	0.10
Mono Calcium Phosphate	1.59	1.41
Choline CL‐60%	0.003	—
Total	100.00	100.00
*Calculated nutrient, %*		
ME, kcal/kg	3000	3100
Crude protein, %	23.0	21.15
Non phytate P, %	0.48	0.44
Calcium, %	0.96	0.87
D‐Lysine, %	1.28	1.15
Sulfur amino acids, %	0.95	0.87
Threonine, %	0.86	0.77
*Proximate composition analysis* ^*c*^ *of the basal diet on a feed basis (g/kg)*		
Moisture	63	71
Dry matter	937	929
Crude ash	60	53
Crude protein	215	192
Crude fat (E.E.)	79	80
Crude fiber	32	29
NFE^d^	551	575

^a^ The vitamin‐mineral premix components per kg: vitamin A, 12,000,000 IU; vitamin D3, 5,000,000 IU; vitamin E, 80,000 IU; vitamin K3, 3200 mg; vitamin B1, 3200 mg; vitamin B_2_, 8600 mg; vitamin B_3_, 65,000 mg; pantothenic acid, 20,000 mg; vitamin B_6_, 4300 mg; biotin, 220 mg; antioxidant (BHA + BHT), 50,000 mg; B_9_, 2200 mg; B_12_, 17 mg.

^b^ Copper, 16,000 mg; iodine, 1250 mg; iron, 20,000 mg; manganese, 120,000 mg; selenium, 300 mg, and zinc, 110,000 mg.

^c^ This analysis was carried out in duplicates.

^d^ NFE: Nitrogen‐free Extract represents the feed's soluble carbohydrates.

### 2.4 Feed Analysis and Bioactive Chemicals of RNFS Probiotic Analysis

Feed samples were analyzed using AOAC (2006) methods to determine proximate composition. RNFS samples were extracted using methanol, filtered, and analyzed using a 6890 N gas chromatography–mass spectrometry (GC–MS) system (Agilent Technologies, Palo Alto, CA, USA) equipped with an HP‐5MS column (30 m × 0.25 mm × 0.25 μm film thickness) and a 5973 N mass selective detector, following Azzam et al. (2020). Bioactive compounds, including phenolic compounds, fatty acid esters, and organic acids were quantified as percentages of the extract. The oven temperature was programmed from 60°C (2 min) to 320°C (20 min) at 6°C/min. Chemical constituents were identified by comparing their mass spectra, retention indices, and compound quality with entries in the National Institute of Standards and Technology (NIST) library using AMDIS software.

### 2.5 Performance Measurements

Individual body weights were recorded on Days 1, 14, and 28 to calculate body weight gain (BWG). Also, feed weights per cage were recorded on Days 1, 14, and 28 to calculate daily feed intake (DFI), allowing calculation of feed conversion ratio (FCR) for each period (Day 0–14 “starter”, 15–28 “grower”, 0–28 “overall”). If any feed spillage occurred, it was collected and subtracted from feed offered. Weights were recorded using a digital balance (±0.01 g precision).

### 2.6 Carcass and Organ Measurements

On Day 28, one broiler per replicate (*n* = 8 per group) was randomly selected for slaughter using a random number table. This sample size is consistent with previously published broiler studies assessing carcass and organ characteristics (Al‐Garadi et al. 2025; Junior et al. 2024; Qaid et al. 2024). All dissections were performed by the same trained researcher, and organ weights were taken fresh. Carcass weights were recorded both immediately after slaughter (hot carcass weight) and after 24 h of chilling at 4°C (cold carcass weight). Carcass yield was calculated as hot carcass weight relative to live weight. The following were weighed individually: breast (with/without bone), legs, wings, proventriculus, heart, kidney, pancreas, liver, and lymphoid organs (bursa of Fabricius, thymus, spleen). Relative weights were computed as a percentage of hot carcass weight (Alqhtani et al. 2023). Giblet weight included gizzard, proventriculus, heart, and liver (excluding gallbladder), and eviscerated carcass weight excluded neck, skin, abdominal fat, and internal organs.

### 2.7 Statistical Analysis

The statistical model followed a completely randomized design using SAS (2012) (v.9.2; Cary, NC, USA), applying one‐way ANOVA:

$$\gamma_{\mathrm{ij}}=\mu +T_{i}+e_{\mathrm{ij}}$$

where *Y*_*ij*_ = observed value; *μ* = overall mean; *T*_*i*_ = treatment effect, and *e*_*ij*_ = residual error.

Data normality was checked via Kolmogorov–Smirnov test. Cage was used as the experimental unit for performance data; individual bird served for carcass traits (*n* = 8). Treatment means were compared using the Ryan–Einot–Gabriel–Welsch Q (REGWQ) test, with significance set at *p* < 0.05. Results are presented as mean ± SEM.

## 3 Results

### 3.1 Phytochemical Composition, Macronutrients, and Total Phenolic Content

The nutrient composition of the grower‐phase basal diet is summarized in Table 1, indicating a high content of macronutrients, particularly crude protein and nitrogen‐free extract. As shown in Table 1, the calculated crude protein levels were 23.0% for the starter diet and 21.15% for the grower diet, aligning with Aviagen recommendations for Ross 308. In comparison with calculated formulation values, proximate analysis of the actual diet batches yielded slightly lower values of 21.5% (215 g/kg) for the starter and 19.2% (192 g/kg) for the grower, which fall within the expected range of analytical variation arising from natural ingredient variability and laboratory differences.

As shown in Table 2, GC–MS profiling identified 45 volatile and semi‐volatile compounds in RNFS, including phenolic derivatives, organic acids, and fatty acid esters, which together indicate a diverse phytochemical composition with potential bioactive properties. The major constituents included trimethyl 1,2,3‐propane‐tricarboxylate (9.52%), phenol (9.17%), and 2,4‐di‐tert‐butylphenol (6.86%), which are notable for their antioxidant and antimicrobial properties. Other prominent compounds included naphthalene derivatives, fatty acid esters such as methyl stearate and methyl linoleate, and silanes, contributing to the functional diversity of the extract. These findings support the presence of phenolic and lipid‐soluble bioactive components, which may underlie the observed biological effects of RNFS in broiler performance and health.

**TABLE 2:** RNFS analysis using GC‐mass spectrometry.

RT (min)	Area	Hit name	%	MW
18.576	3,003,457	Trimethyl 1,2,3‐propanetricarboxylate	9.52	218.079
8.122	2,892,759	Phenol	9.17	94.042
20.631	2,163,619	2,4‐Di‐tert‐butylphenol	6.86	206.167
13.678	1,735,143	Naphthalene, 2,3,6‐trimethyl—	5.50	170.11
12.746	1,577,819	Silane, butyltrimethyl—	5.00	130.118
31.125	1,574,963	11‐Octadecenoic acid, methyl ester	4.99	296.272
12.419	1,445,307	1H‐Pyrazole, 1,5‐bis—	4.58	212.117
28.344	1,310,078	Hexadecanoic acid, methyl ester	4.15	270.256
9.518	1,193,730	Butanedioic acid, dimethyl ester	3.79	146.058
31.966	1,104,075	1‐Octadecanol	3.50	342.332
16.751	950,612	2‐Pentenedioic acid, 2‐methoxy‐, dimethyl ester	3.01	188.068
6.652	730,189	Anisole	2.32	108.058
3.985	651,549	Allyl alcohol	2.07	130.081
31.016	329,904	9,12‐Octadecadienoic acid (Z, Z)‐, methyl ester	2.05	294.256
20.178	622,080	trans‐1,2‐ethylene	1.97	172.11
37.167	604,587	Bis(2‐ethylhexyl) phthalate	1.92	390.277
31.531	561,298	Methyl stearate	1.78	298.287
30.261	556,769	Palmitic Acid	1.77	328.28
5.21	555,794	Isobutanol	1.76	146.113
35.284	552,144	Hexanedioic acid, bis (2‐ethylhexyl) ester	1.75	370.308
33.236	534,082	Stearic acid	1.69	356.311
13.793	489,799	Propylene glycol	1.55	220.131
27.989	473,636	7,9‐Di‐tert‐butyl‐1‐oxaspiro (4,5) deca‐6,9‐diene‐2,8‐dione	1.50	276.173
22.576	402,060	Ethoxyacetic acid	1.28	176.087
29.477	395,841	3‐Eicosene, (E)—	1.26	280.313
7.138	395,053	Ethanimidic acid	1.25	203.116
5.822	384,120	Acetoin	1.22	160.092
15.589	329,973	Benzenepropanoic acid, methyl ester	1.05	164.084
27.234	309,789	Phthalic acid, isobutyl nonyl ester	0.98	348.23
28.401	298,270	Benzenepropanoic acid, 3,5‐bis (1,1‐dimethylethyl)‐4‐hydroxy‐, methyl ester	0.95	292.204
17.724	259,676	Benzenepropanoic acid, alpha. ‐hydroxy‐, methyl ester	0.82	180.079
32.567	257,127	1‐Hexacosene	0.82	364.407
8.981	239,941	Benzohydroxamic acid	0.76	281.127
22.284	220,409	Benzoic acid, 3,4‐dimethoxy‐, methyl ester	0.70	196.074
5.416	220,318	2‐Pentanol	0.70	160.128
9.959	208,307	Phenmetrazine	0.66	249.155
14.256	194,069	Dimethyl 3‐hydroxy‐3‐methylpentane‐1,5‐dioate	0.62	190.084
13.478	187,185	Lactic Acid	0.59	234.111
17.501	180,781	Nonanoic acid	0.57	230.17
7.521	175,576	2‐Pentenal, 2‐methyl—	0.56	98.073
15.818	159,708	1,3‐Bis‐propene	0.51	186.126
16.288	154,326	Catechol	0.49	338.21
8.368	139,997	Methoxyacetic acid	0.44	204.118
16.997	139,387	Tartronic acid	0.44	336.124
14.891	119,191	Ethylene glycol	0.38	206.116
11.641	117,149	4‐Hydroxybutanoic acid	0.37	248.126
24.075	115,583	Adipic acid, butyl hexyl ester	0.37	286.214
Total			100.00	

### 3.2 Performance Indices

Growth performance parameters for broilers were presented in Table 3. Broilers supplemented with 0.2% RNFS exhibited significantly (*p =* 0.04; SEM ±27.1 g) greater average body weight (ABW) at Day 28 compared to T0, T4, and 5 groups. This dosage also led to significant improvements in BWG during Days 14–28 (*p* = 0.05; SEM ±1.93 g) and a numerical improvement in overall BWG (*p* = 0.07; standard error ±0.96 g). DFI and FCR were not significantly affected by treatment (*p* > 0.05).

**TABLE 3:** Effect of RISCO–NUTRIFOUR probiotic on growth performance parameters in broilers during starter and grower phases.

Parameters	Treatments*						SEM	*p*
	T0	T1	T2	T3	T4	T5		
*Average body weight (ABW; g/bird)*								
D 1	41.4	41.5	41.7	41.7	41.4	41.8	0.082	0.106
D 14	438.7	443.3	427.4	421.0	415.2	433.6	9.33	0.276
D 28	1449.7^b^	1503.0^ab^	1557.5^a^	1474.0^b^	1444.7^b^	1513.0^ab^	27.08	0.041
*Average daily weight gain (DWG; g/bird/day)*								
D 1–D 14	28.4	28.7	27.5	27.1	26.7	28	0.622	0.126
D 14–D 28	72.2^b^	75.7^ab^	80.7^a^	75.2^ab^	73.5^ab^	77.1^ab^	1.93	0.056
D 1–D 28	50.3	52.2	53.8	51.2	50.1	52.5	0.967	0.077
*Average daily feed intake (DFI; g/bird/day)*								
D 1–D 14	33.3	34.4	34.2	33.1	34.7	34.1	0.883	0.775
D 14–D 28	108.8	114.7	115.6	117.3	109.2	118.8	3.21	0.159
D 1–D 28	71.1	74.6	74.9	75.2	72.0	76.5	1.631	0.179
*Feed conversion (FCR; g: g/bird/day)*								
D 1–D 14	1.18	1.20	1.24	1.22	1.30	1.22	0.039	0.225
D 14–D 28	1.51	1.52	1.43	1.56	1.49	1.54	0.042	0.394
D 1–D 28	1.41	1.43	1.40	1.47	1.44	1.46	0.031	0.458

Abbreviation: SEM, standard error of the mean.

* Treatments were administered via the basal diet or drinking water as follows: T0—no probiotic supplementation (0.0% RNFS); T1–T3: RNFS at 0.1%, 0.2%, and 0.4%, respectively; T4: *B. subtilis* at 0.1%; and T5: *S. cerevisiae* at 0.1%. *n* = 8 replicated cages per treatment. ^a&b^Within the same row, means with different superscripts show significant differences (*p* < 0.05).

### 3.3 Carcass and Organ Characteristics

Data on slaughter weight, hot and cold carcass weights, carcass yield, and absolute and relative organ weights of broilers at Day 28 are detailed in Tables 4 and 5. No significant differences (*p* > 0.05) were observed among treatment groups in carcass characteristics. However, a significant difference was detected in the absolute gizzard weight (*p* = 0.023). The gizzard was heaviest in broilers supplemented with 0.2% RNFS and 0.1% *S. cerevisiae* , lightest in the T0 group, and intermediate in the remaining treatments (Table 4).

**TABLE 4:** Effect of RISCO–NUTRIFOUR probiotic on absolute (g) and relative (% relative to hot carcass weight) carcass trait weights of broilers at 28 days of age.

Parameters	Treatments*						SEM	*p*
	T0	T1	T2	T3	T4	T5		
*Carcass characteristics*								
Slaughter weight (g)	1490	1635	1620	1548	1537	1571	46.4	0.334
Hot carcass weight (g)	928	1065	963	942	944	967	38.6	0.168
Cold carcass weight (g)	898	950	926	888	916	912	30.3	0.773
Carcass yield (%)	62.2	65.2	59.6	60.9	61.4	61.6	1.78	0.577
*Absolute weight (g)*								
Breast	449	468	457	436	434	428	19.70	0.690
Breast without bone	288	300	307	293	296	310	15.72	0.888
Leg	383	399	394	380	398	400	11.50	0.720
Wing	74.6	80.3	72.9	81.1	80.9	80.5	3.03	0.173
Gizzard	35.5^b^	42.9^ab^	43.5^a^	39.1^ab^	40.1^ab^	44.8^a^	2.02	0.023
Proventriculus	6.93	8.04	7.07	7.16	6.95	7.44	0.385	0.202
Liver	30.4	33.5	34.5	33.7	29.9	32.4	2.28	0.672
Heart	8.75	9.17	9.67	9.11	8.78	9.5	0.466	0.597
Kidney	7.54	8.94	8.58	7.78	7.03	9.09	0.530	0.227
Pancreas	4.47	4.48	4.67	4.14	4.19	4.57	5.12	0.532
Giblets**	81.6	93.7	94.8	89.1	85.6	94.2	5.31	0.690
*Relative weight (%)*								
Breast	48.1	44.5	47.4	46.3	45.9	44.1	1.21	0.117
Breast without bone	30.8	28.7	31.9	31	31.3	31.8	2.20	0.481
Leg	41.4	38.1	40.9	40.4	42.2	41.5	2.53	0.333
Wing	7.99	7.68	7.60	8.61	8.59	8.33	1.06	0.471
Gizzard	3.87	4.14	4.53	4.14	4.28	4.65	0.346	0.755
Proventriculus	0.756	0.768	0.736	0.757	0.739	0.769	0.056	0.888
Liver	3.28	3.22	3.59	3.58	3.17	3.36	0.226	0.392
Heart	0.939	0.882	1.01	0.963	0.935	0.988	0.043	0.533
Kidney	0.822	0.851	0.893	0.825	0.749	0.942	0.058	0.490
Pancreas	0.482	0.432	0.486	0.44	0.445	0.476	1.98	0.554

Abbreviation: SEM, standard error of the mean.

* Treatments were administered via the basal diet or drinking water as follows: T0 – no probiotic supplementation (0.0% RNFS); T1–T3: RNFS at 0.1%, 0.2%, and 0.4%, respectively; T4: *B. subtilis* at 0.1%; and T5: *S. cerevisiae* at 0.1%. *n* = 8 per treatment. ^a&b^Within the same row, means with different superscripts show significant differences (*p* < 0.05).

** Giblets (Gizzard, proventriculus, heart, and liver without gallbladder).

**TABLE 5:** Effect of RISCO–NUTRIFOUR probiotic on absolute (g) and relative (% relative to hot carcass weight) weights of lymphoid organs in broilers, assessed at day 28.

Parameters	Treatments^a^						SEM	*p*
	T0	T1	T2	T3	T4	T5		
*Absolute weights (g)*								
Thymus	7.08	6.20	6.46	5.73	4.83	5.73	0.684	0.226
Bursa of Fabricius	3.33	4.14	3.32	3.31	3.33	2.94	0.34	0.265
Spleen	1.30	1.39	1.11	1.25	1.24	1.15	1.89	0.517
*Relative weight (%)*								
Thymus	0.764	0.538	0.674	0.604	0.513	0.600	0.078	0.111
Bursa of Fabricius	0.359	0.398	0.342	0.353	0.355	0.262	0.035	0.386
Spleen	0.141	0.132	0.115	0.133	0.132	0.120	0.189	0.562

Abbreviation: SEM, standard error of the mean.

^a^ Treatments were administered via the basal diet or drinking water as follows: T0 – no probiotic supplementation (0.0% RNFS); T1–T3: RNFS at 0.1%, 0.2%, and 0.4%, respectively; T4: *B. subtilis* at 0.1%; and T5: *S. cerevisiae* at 0.1%. *n* = 8.

## 4 Discussion

Supplementation with single‐strain probiotics ( *B. subtilis* or *S. cerevisiae*), or multi‐strain probiotics, resulted in enhanced growth performance, aligning with findings by Abdel‐Latif et al. (2018), who reported that combined supplementation of *Clostridium butyricum* and *S. cerevisiae* (0.25 g/kg each) was more effective than individual strains in improving performance, immunity, and gut health in broilers.

Our findings are also consistent with Bostami et al. (2016), who documented similar dietary growth‐promoting effects of multi‐strain probiotics, comparable to antibiotic treatments. Bostami et al. (2016) used a similar dietary multi‐strain probiotic except *Candida ethanolic*, similar to our study. Additionally, Izadi et al. (2025) highlighted that probiotic blends based on *Enterococcus faecium* could serve as antibiotic‐free alternatives in poultry diets.

*B. subtilis* and *S. cerevisiae* are widely utilized in poultry production. *B. subtilis* , in particular, is recognized as a non‐pathogenic, Gram‐positive probiotic capable of replacing antibiotics in broiler diets (Abd El‐Hack et al. 2020; Bai et al. 2017). According to Chen et al. (2009), improved growth from Days 0 to 21 in broilers fed fermented feed was attributed to *B. subtilis* inclusion rather than the added moisture. Guo et al. (2024) also noted that protease production by *B. subtilis* enhanced nutrient utilization, particularly in cottonseed meal‐based diets.

Performance improvement was likely due to the synergistic effect of dual‐stage fermentation involving both *B. subtilis* and *S. cerevisiae* rather than isolated factors like water addition or single‐strain fermentation (Chen et al. 2009). In fact, Chen et al. (2009) reported reduced ABW, BWG, and DFI in water‐added groups compared to fermented feed groups. Varying *B. subtilis* dosages consistently enhanced BWG across several studies (Al‐Marzooqi et al. 2025; Bai et al. 2017; Khan et al. 2025; Mohamed et al. 2022). Amerah et al. (2013) found that a diet supplemented with 1.5 × 10^8^ CFU/kg of *B. subtilis* significantly increased average daily BWG. Similarly, Mohamed et al. (2022) showed that *B. subtilis* (3 × 10^8^ CFU/g) improved BWG and the weight of the bursa of Fabricius at 21 days.

Both *B. subtilis* and *S. cerevisiae* support gut health by establishing favorable environments for beneficial anaerobic microbes such as Lactobacillus spp., protecting intestinal villi from pathogenic invasion (He et al. 2019; Laptev et al. 2019; Teng and Kim 2018). These probiotics also enhance immune response, nutrient retention, digestive enzyme activity, and production of growth‐promoting metabolites (Fazelnia et al. 2021).

Probiotic‐supplemented broilers often perform comparably to those receiving antibiotics, as probiotics facilitate gut colonization by beneficial microbes (Al‐Sagan and Abudabos 2018; García‐Reyna et al. 2023; Hussein et al. 2020). These microbes reduce pathogen attachment via competitive exclusion (Abudabos et al. 2013; Murshed et al. 2024; Vieco‐Saiz et al. 2019) and enhance amino acid and mineral absorption (Poberezhets et al. 2021).

*B. subtilis* exhibits antimicrobial effects against pathogens like *Clostridium perfringens* ( *C. perfringens* ) through immune modulation (Olson et al. 2021; Yurong et al. 2005). Murshed et al. (2024) found that probiotic supplementation inhibited *C. perfringens* proliferation in the ileum via competitive exclusion. According to Teo and Tan (2006), *B. subtilis* also produces acetic and lactic acid from glucose, which supports beneficial microbial colonization. Such probiotics, including yeasts, help maintain gut homeostasis and improve nutrient efficiency by modulating microbial balance (Murshed et al. 2024).

Numerous studies have confirmed the efficacy of *B. subtilis* and *S. cerevisiae* in enhancing growth performance, feed conversion, and pathogen resistance (Bahaddad et al. 2023; Paryad and Mahmoudi 2008). In the present study, a 0.2% inclusion rate of RNFS significantly improved broiler ABW and BWG from Days 14–28. This group outperformed the *B. subtilis* , negative control, and *S. cerevisiae* groups in ABW by 7.16%, 6.79%, and 2.33%, respectively, and in BWG by 11.77%, 9.80%, and 4.67%, respectively. Bahaddad et al. (2023) similarly observed improved growth, FCR, and gut histology following dietary Bacillus supplementation, supporting better digestion and nutrient absorption (Jayaraman et al. 2013).

Although inclusion of 1.5% *S. cerevisiae* has been shown to enhance performance and carcass traits (Paryad and Mahmoudi 2008; Okasha et al. 2023) reported no significant effect on production or carcass characteristics. In our study, carcass weight and internal organ weights (heart, liver, kidney, proventriculus, pancreas, lymphoid tissues) did not differ significantly among treatments. The sample size used for organ‐level outcomes (*n* = 8 per group) may have limited the ability to detect subtle differences among treatments. However, this number is consistent with previously published broiler studies evaluating carcass and organ traits (Al‐Garadi et al. 2025; Junior et al. 2024; Qaid et al. 2024). Moreover, the improvement in growth performance without corresponding changes in carcass trait weights may be explained by differences in the physiological drivers of these outcomes. RNFS Probiotics, particularly at 0.2%, may enhance nutrient digestibility, gut health, and feed efficiency, leading to better BWG and FCR. However, carcass composition traits, including organ and muscle weights, are influenced not only by nutrient utilization but also by genetic potential, slaughter age, and metabolic partitioning of nutrients (Bai et al. 2024; Li et al. 2025; Zhang et al. 2024). Since birds were slaughtered at 28 days, the trial may not have captured longer‐term effects on carcass yield that often become more apparent in later growth phases. Similar findings have been reported previously, where probiotics improved growth performance and feed efficiency but did not consistently alter carcass traits or lymphoid organs at comparable ages (Murshed et al. 2024; Sarangi et al. 2016; Silva et al. 2018). However, Tang et al. (2021) found improved carcass traits with *B. subtilis* supplementation, and Park and Kim (2014) observed increased bursa weight. Other studies (Al‐Sagan et al. 2020; Alkhalf et al. 2010; Awad et al. 2009) also reported increases in lymphoid organ weights due to probiotic use. Thus, the discrepancy likely reflects both the short trial duration and the multifactorial regulation of carcass traits compared to growth performance.

Nevertheless, not all studies report consistent outcomes. Reis et al. (2017) found no impact of *B. subtilis* on liver and spleen weights. Hatab et al. (2016) reported no effect on gizzard, heart, pancreas, or liver weights, while others (Hatab et al. 2016; Zhang et al. 2012) showed variable results for the bursa. These inconsistencies may stem from differences in probiotic strains, doses, broiler genetics, diet composition, or environmental factors (Abdel‐Latif et al. 2018).

The positive impacts of RNFS on broiler performance—particularly the significant improvement in ABW and numerical enhancement in FCR at the 0.2% inclusion level—can be partly attributed to its diverse phytochemical composition. GC–MS analysis revealed the presence of several bioactive compounds, notably phenolic compounds such as phenol and 2,4‐di‐tert‐butylphenol, known for their antioxidant and antimicrobial properties. Additionally, compounds like trimethyl 1,2,3‐propane‐tricarboxylate, fatty acid esters (e.g., methyl stearate, methyl linoleate), and naphthalene derivatives contribute to gut modulation, nutrient absorption, and immune regulation. Although phenol and naphthalene derivatives were detected, their low concentrations are within safe ranges for poultry diets, consistent with prior safety evaluations (EFSA 2012). These constituents likely played a synergistic role in enhancing nutrient utilization, maintaining gut integrity, and promoting metabolic efficiency in broilers, which explains the observed performance benefits without compromising carcass quality or lymphoid organ development.

This study delivers the potential of RNFS as a multi‐strain, multi‐genera probiotic for improving broiler performance. Its practical two‐phase supplementation approach and comprehensive evaluation of growth and carcass traits are commendable. However, the mechanistic insights, such as microbiome profiling and antioxidant markers, were not addressed. Future studies should explore these aspects and validate results under varied conditions to support broader application in the poultry industry.

## 5 Conclusion

In conclusion, the findings suggest that dietary RISCO‐NUTRIFOUR at a 0.2% dosage improved broiler growth performance and BWG from Days 14 to 28, and may serve as a sustainable alternative to antibiotics. During Days 14–28, RNFS supplementation at 0.2% improved daily body weight gain (BWG) by 4.67%, 9.80%, and 11.77% relative to *S. cerevisiae* , *B. subtilis* , and the negative control groups, respectively. Additionally, the 0.2% dosage improved absolute gizzard weight. Further studies should investigate these effects at the microbiota and molecular level to confirm mechanisms.

## Author Contributions

**Abdulaziz A. Al‐abdullatif:** supervision, resources, software, data curation, and validation. **Maged A. Al‐Garadi:** conceptualization, project administration, supervision, visualization, funding acquisition, resources, and validation. **Mohammed M. Qaid:** conceptualization, software, validation, investigation, data curation, methodology, writing – original draft preparation. **Abdulkareem M. Matar:** formal analysis, and data curation. **Mohammed Al‐Badwi:** data curation, validation. **Mohsen M. Alobre:** data curation, software. **Gamaleldin M. Suliman:** conceptualization, data curation. **Elsayed O. Hussein:** data curation, software. All authors have writing‐review and editing. All authors have read and agreed to the published version of the manuscript.

## Funding

This work was supported by the Ongoing Research Funding Program (ORF‐2025‐690), King Saud University, Riyadh, Saudi Arabia.

## Conflicts of Interest

The authors declare no conflicts of interest.

## Data Availability

The data that support the findings of this study are available from the corresponding author upon reasonable request.
